# High HIV viral suppression among adults receiving WHO-recommended first-line dolutegravir-based antiretroviral therapy in low- and middle-income countries: a systematic review and meta-analysis of programmatic evidence

**DOI:** 10.1186/s12981-025-00788-8

**Published:** 2025-09-26

**Authors:** Amalia Girón-Callejas, Rolando Lorenzana, Michael Pickles, Seth Inzaule, Michael R. Jordan, Sheilee Diaz, Charlotte Vrinten

**Affiliations:** 1 School of Public Health, Imperial College London, 90 Wood Lane, W12 0BZ London, UK; 2 ICAP, Columbia University, New York, USA; 3 Medical Research Council Centre for Global Infectious Disease Analysis, School of Public Health, Imperial College London, London, UK; 4 HIV Prevention Trials Network Modelling Centre, Imperial College London, London, UK; 5 Department of Global Health, Amsterdam Institute for Global Health and Development, Amsterdam UMC, University of Amsterdam, Amsterdam, The Netherlands; 6 Division of Geographic Medicine and Infectious Diseases, Tufts Medical Center, Boston, MA USA; 7 Department of Public Health and Community Medicine, Tufts University School of Medicine, Boston, USA; 8 Center for Global Health and Tropical Medicine, Instituto de Higiene e Medicina Tropical, Universidade Nova de Lisboa, Lisbon, 1349-008 Portugal; 9 Centro de Estudios en Salud, Universidad del Valle de Guatemala, Guatemala, Guatemala

**Keywords:** HIV, Dolutegravir, Antiretroviral therapy, Highly active, Viral load, Developing countries, Systematic review

## Abstract

**Background:**

This systematic review and meta-analysis assessed viral suppression among adults receiving WHO-recommended first-line dolutegravir-based ART in programmatic settings in low- and middle-income countries (LMICs).

**Methods:**

A systematic search of Ovid MEDLINE, Embase, and major HIV conferences (IAS, AIDS, and CROI) from January 2019 to September 2024 identified cohort and cross-sectional studies reporting viral suppression among adults receiving WHO-recommended first-line dolutegravir-based ART in LMICs. Studies with follow-ups ≤ 4 months or using non-WHO-recommended regimens were excluded. Pooled estimates were calculated using random-effects meta-analysis. Sensitivity analyses excluded outliers. Subgroup analyses distinguished adults initiating versus transitioning to dolutegravir-based ART. Both on-treatment and intention-to-treat outcomes were assessed.

**Results:**

Twenty-two studies (*n* = 47 to 50,742) from 13 countries were included. On-treatment pooled viral suppression was 95% (95% CI: 91–97%, I²= 96%) at six months, 96% (94–98%, I² = 97%) at 12 months, and 98% (96–99%, I² = 94%) at 24 months. Sensitivity analysis removing outliers decreased heterogeneity and slightly lowered the 6‑month estimate (to 94%), with negligible change at 12 months. At 6 months, viral suppression was higher in those transitioning than initiating ART (98% vs. 94%, *p* < 0.01), with similar rates at 12 months (97%, *p* = 0.67). The pooled intention-to-treat 12-month viral suppression rate was 89% (82–93%, I² = 95%), with no significant difference by ART status (initiating 86% vs. transitioning 91%, *p* = 0.44).

**Conclusion:**

Adults retained in care receiving WHO-recommended first-line dolutegravir-based ART achieved viral suppression rates of ≥ 95% up to two years. These findings align with the UNAIDS 95% suppression target and reinforce the role of dolutegravir-based regimens in ending HIV as a public health threat.

*Trial registration:* CRD42024557769.

**Supplementary Information:**

The online version contains supplementary material available at 10.1186/s12981-025-00788-8.

## Background

HIV infection remains a global public health challenge, with an estimated 39.9 million people living with the virus and 1.3 million new infections reported in 2023 [1]. In low- and middle-income countries (LMICs), HIV-related deaths rank as the fourth leading cause of mortality from communicable diseases, contributing to 2.25% (1.95–2.65%) of total disability-adjusted life years [2]. Antiretroviral therapy (ART) has demonstrated efficacy in suppressing HIV replication, enabling individuals to live longer and healthier lives while also reducing HIV transmission [3, 4]. By 2023, 30.7 million people worldwide were receiving ART [1].

At the end of 2018, the World Health Organization (WHO) recommended dolutegravir-based ART as the preferred first-line regimen for adults [5]. This recommendation was based on evidence from clinical trials demonstrating superior viral suppression rates and reduced rates of treatment discontinuation compared with efavirenz-based regimens [6]. WHO also advised that people living with HIV already receiving a first-line non-nucleoside reverse transcriptase inhibitor-containing regimen should be transitioned to DTG-based ART, with appropriate consideration of viral load monitoring, adherence support, and the clinical context [5]. According to WHO, by mid-2022, 108 of 123 reporting countries had adopted dolutegravir-based ART as their preferred first-line treatment protocol [7]. The 2024 Clinton Health Access Initiative HIV Market Report indicated that approximately 95% of adults receiving treatment in generic-accessible LMICs receive a DTG-based regimen [8]. As the global transition to DTG-based ART continues, evaluating its effectiveness in real-world, programmatic settings is critical to inform the performance of ART programmes.

Achieving the Joint United Nations Programme on HIV/AIDS (UNAIDS) target of 95% viral suppression by 2030 is crucial for ending the HIV epidemic as a public health concern [9, 10]. This target aligns with the Sustainable Development Goal 3, which calls for ending the AIDS epidemic by 2030 as part of broader efforts to ensure healthy lives and promote well-being for all [11]. Therefore, monitoring viral suppression among individuals living with HIV is crucial for tracking progress toward this global health goal and serves as a key indicator of the success of HIV treatment and prevention efforts. While dolutegravir-based ART has shown high efficacy in clinical trials, its effectiveness in achieving viral suppression in real-world contexts remains uncertain, especially in LMICs where challenges with treatment adherence and retention in care are prominent [12, 13].

Although observational studies on the clinical outcomes of dolutegravir-based ART in LMICs have emerged, no systematic review has yet synthesised these findings. This systematic review and meta-analysis addresses this gap by estimating the prevalence of HIV viral suppression among adults receiving WHO-recommended first-line dolutegravir-based ART regimens in programmatic settings in LMICs at different time points.

## Methods

This systematic review adhered to the Preferred Reporting Items for Systematic Reviews and Meta-Analyses (PRISMA) guidelines (Supplementary Material 1 and 2) [14]. The review protocol was registered in the International Prospective Register of Systematic Reviews (PROSPERO registration number CRD42024557769) [15].

### Search strategy and study selection

A literature search was performed via Ovid Embase and MEDLINE (Supplementary Material 3), which included terms for HIV, dolutegravir, viral suppression, and LMIC as defined by the World Bank [16]. The search was limited to English-language biomedical literature published between January 2019 and September 2024. The start date was chosen to capture outcomes following the WHO’s December 27, 2018, recommendation for a dolutegravir-based first-line ART regimen [17]. Additionally, online conference abstracts from IAS, AIDS and CROI (2019–2023) were manually reviewed to capture recent programmatic data. The final database search was conducted on September 27, 2024, and the conference abstract screening was completed on September 28, 2024.

**Fig. 1:**
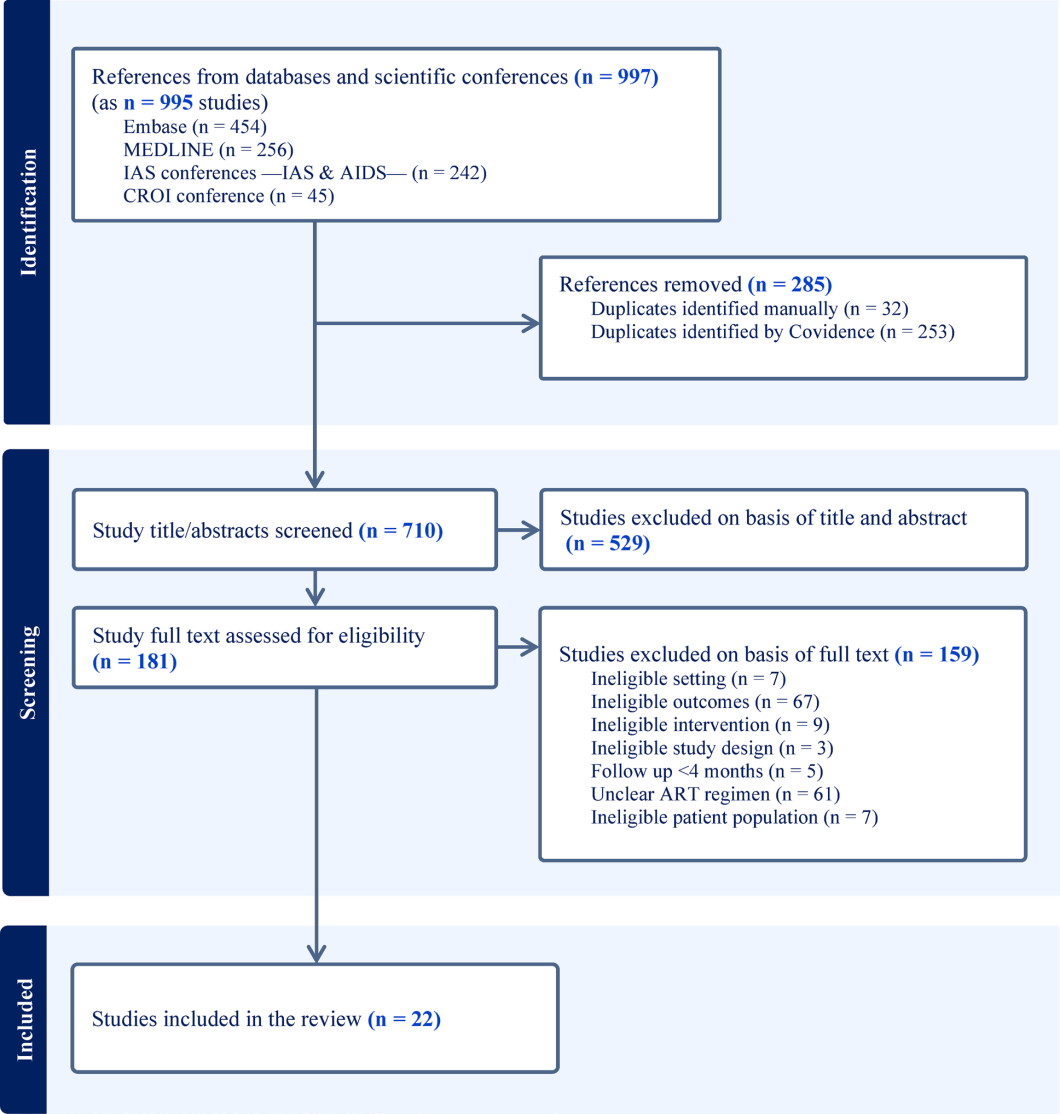
PRISMA flow chart of study selection. Flow chart shows the process of study selection for the systematic review. When multiple references reported on the same cohort at different time points, they were consolidated into a single study, resulting in a higher number of reported references than included studies. *ART* antiretroviral therapy, *CROI* Conference on Retroviruses and Opportunistic Infections, *IAS* International AIDS Society

Two independent reviewers used Covidence [18] for deduplication and study selection. Records were screened by title and abstract, and the full texts of the remaining records were reviewed for inclusion. When multiple references described the same study (e.g., at different time points or in separate publications), they were consolidated into a single study for analysis. Consequently, the number of included references exceeded the number of unique studies, as reflected in the PRISMA flow diagram (Fig. 1). Discrepancies were resolved through discussion between the two reviewers.

This review included original research papers and conference abstracts reporting observational studies (cross-sectional or cohort) on adults living with HIV in LMICs receiving WHO-recommended first-line ART with dolutegravir in programmatic settings [5]. Programmatic settings were defined as routine HIV service delivery environments, including public sector facilities and NGO-supported sites, operating outside the context of controlled clinical trials. Adults were defined as individuals aged ≥ 19 years [5]. Studies with ≥ 90% adult participants were included. LMICs were defined per the World Bank’s 2024 gross national income per capita [16]. Eligible studies reported the proportion of viral suppression at specified time points after starting or transitioning to WHO-recommended first-line dolutegravir-based ART.

The review focused on studies prescribing a WHO-recommended first-line ART regimen that includes dolutegravir [5]. Eligible regimens were (a) tenofovir disoproxil fumarate with lamivudine and dolutegravir; (b) tenofovir disoproxil fumarate with emtricitabine and dolutegravir; (c) tenofovir alafenamide with lamivudine and dolutegravir; (d) tenofovir alafenamide with emtricitabine and dolutegravir; and (e) abacavir with lamivudine and dolutegravir. Studies not using WHO-recommended ART regimens and those with follow-up periods of less than four months were excluded.

### Data extraction

Two reviewers independently used Covidence [18] to collect data on the study setting, design, time frame, number of participants, sex distribution, descriptive age statistics at dolutegravir-based ART initiation, participant type (initiating with vs. transitioning to dolutegravir-based ART), ART regimen, viral suppression definition (HIV RNA threshold in copies/ml), number of participants with and without viral suppression, and analysis type (on-treatment vs. intention-to-treat). If the number of participants with and without viral suppression was not explicitly reported, it was manually calculated from the available data. In cases where studies documented viral suppression at several time points or applied multiple criteria to define viral suppression, all reported virological outcomes were extracted.

The classification of on-treatment and intention-to-treat analyses was based on the analytic approach used in each study. On-treatment analyses referred to estimates restricted to individuals retained in care with available viral load results at the specified time point. Intention-to-treat analyses included all individuals who initiated or transitioned to dolutegravir-based ART, with those lost to follow-up, transferred, or missing viral load data retained in the denominator and considered not virally suppressed in the numerator. These definitions were extracted as reported and not modified by the review team.

### Statistical analysis

Random-effects meta-analyses were conducted to estimate the pooled prevalence and 95% confidence interval (95% CI) of viral suppression. Between-study variance (τ²) was calculated using the restricted maximum likelihood method. Prevalence data were logit-transformed to stabilise variances and back-transformed to the original scale for presentation. Both common-effect and random-effects weights were calculated for each study, although the primary analysis and interpretation focused on the random-effects model due to anticipated heterogeneity among studies. The forest plots present common and random effects weights and summary estimates, providing additional context for comparison. Analyses were stratified by on-treatment and intention-to-treat approaches, based on the classification reported by each study. Meta-analyses were performed using R (version 4.2.2) and the meta package [19, 20].

The pooled prevalence of viral suppression was calculated for each duration of dolutegravir-based ART (i.e., 6, 12, and 24 months) using the threshold equal to or closest to 1000 copies/ml, which aligns with current WHO standards for viral suppression [5, 21] and the UNAIDS indicator for monitoring progress toward the 95% viral suppression target by 2030 [22]. Studies conducted across multiple countries that reported outcomes by country were treated as separate cohorts, and studies reporting different participant types (e.g., newly initiating vs. transitioning to a dolutegravir-based regimen) were also considered distinct cohorts. Consequently, the total number of reported cohorts exceeded the total number of included studies.

Pre-specified sensitivity and subgroup analyses were conducted to explore potential sources of heterogeneity. First, potential outliers were identified using the find.outliers function from the dmetar R package, which defines outliers as studies whose 95% confidence intervals lie entirely outside the 95% confidence interval of the pooled effect estimate [23]. These outliers were removed, and the random-effects meta-analysis was rerun to assess their impact on the pooled results. Second, a viral load threshold-restricted sensitivity analysis was performed to assess the impact of stricter viral load suppression definitions on pooled prevalence estimates. This analysis included only studies that reported ≤ 1,000 copies/ml as one of the viral load suppression thresholds and for which the ≤ 1,000 threshold was used in the meta-analysis. Studies that reported only stricter thresholds (e.g., ≤ 50, ≤150, or ≤ 400 copies/ml) were removed, and the random-effects meta-analysis was rerun using this restricted dataset. For the subgroup analysis, studies were categorised into “Initiating ART” (adults initiating ART with a dolutegravir-based regimen, with or without previous exposure to ARV drugs) and “Transitioning ART” (adults receiving ART transitioning to a dolutegravir-based ART) groups. Random effects meta-analyses were subsequently performed, and subgroup differences were evaluated via chi-square tests.

### Certainty of evidence

The risk of bias was evaluated with the Joanna Briggs Institute Critical Appraisal Tools for cohort and prevalence studies [24, 25]. Inconsistency was assessed by examining study heterogeneity, and the precision of the pooled estimates was systematically assessed. Publication bias was not analysed, as it may be unreliable in the presence of high heterogeneity (I² ≥ 75%) [23]. A formal GRADE assessment was conducted for the two primary outcomes: 12-month viral suppression under on-treatment and intention-to-treat analyses.

## Results

After removing duplicates, 710 studies were identified as potentially eligible. Title and abstract screening excluded 529 studies (Fig. 1). Among the remaining 181 studies, 22 met the inclusion criteria: three cross-sectional, 12 prospective cohort, and seven retrospective cohort studies (Table 1). Of these, 5 studies (22.7%) were identified from scientific conference abstracts, while the remaining 17 were peer-reviewed journal publications. No preprints were identified among the included studies. These studies were conducted across 13 countries, with Brazil contributing the most (5 studies), followed by Ethiopia, India and Malawi (3 studies each). The total number of study participants ranged from 47 to 50,742, with a female representation varying widely from 4.3 to 70.6%. Fifteen studies reported 6-month viral suppression outcomes, 15 at 12 months, three at 24 months, and one at ≥ 36 months. The viral suppression thresholds varied among studies, with definitions including ≤ 1000, ≤400, ≤ 200, ≤150, ≤ 50 and ≤ 20 copies/ml, with the majority (11 of 22) using ≤ 1000 copies/ml, which was consistent with WHO guidelines. Sixteen studies used on-treatment analysis, three utilised intention-to-treat, and three reported both. The predominant WHO-recommended ART regimen utilised across the studies was tenofovir disoproxil fumarate with lamivudine and dolutegravir. Two studies (Buju 2022 and Marc 2023) were conducted during armed conflicts [26, 27], and one (Brites 2022) included patients with advanced HIV disease [28].

**Table 1:** Characteristics of studies included in the analysis

Study ID	Country	Number of sites	Type of patients ^†^	Viral suppression definition (copies/ml)	Total number of participants	Females (%)	Age in years	Dolutegravir-based first-line ART regimen	Analysis time point (months)	Type of analysis
*Prospective cohort studies*										
Brites 2022 [28]	Brazil	5	Initiating ART	≤ 200; ≤50	92	31.5	Mean 39.4 (SD: 4.7)	100% TLD	12	ITT
Kityo 2022 [54]	Zimbabwe; Haiti; Uganda; Malawi; Kenya; South Africa	13	Initiating ART	≤ 1000; ≤200; ≤50	179	42.0	Median 35 (IQR: 28–42)	100% TLD	6	OT
Marc 2023 [27]	Haiti	1	Initiating ART	≤ 200	246	45.0	Median 39 (IQR: 33–47)	100% TLD	6; 12	OT
Buju 2022 [26]	Democratic Republic of the Congo	NR	Any	≤ 50	468	69.4	Mean 39.0 (SD: 11.9)	100% TLD	6; 12	OT
Schramm 2022 [31]	Malawi	3	Transitioning ART	≤ 1,000; ≤50	1,892	50.1	Male: Median 46 (IQR: 40–54) Female: Median 53 (IQR: 48–59)	100% TLD	6; 12	OT & ITT
Gemechu 2023 [55]	Ethiopia	15	Initiating ART	≤ 1000	235	70.6	Mean 33.9 (SD: 12.1)	98.6% TLD; 1.4% ABC + 3TC + DTG	6	OT
McCluskey 2024 [30, 56, 57]	Uganda	1	Transitioning ART	≤ 1000; ≤ 200; ≤50	500	41.0	Median 47 (IQR: 40–53)	100% TLD	6; 12	OT & ITT
McCluskey 2024 [39]	South Africa	4	Transitioning ART	≤ 50	499	80.0	Median 39 (IQR: 32–50)	100% TLD	6; 12	ITT
Gebremedhin 2024 [32]	Ethiopia	NR	Initiating ART	≤ 50	109	48.6	Median 32 (IQR: 26–40)	100% TDF + 3TC + DTG or TDF + FTC + DTG	6	OT
Kouamou 2024 [58]	Zimbabwe	1	Initiating ART	≤ 1000; ≤50	172	54.0	Median 39 (IQR: 29–48)	100% TLD	6	OT
Sengupta 2023 [59]	India	1	Initiating ART	≤ 1000	376	26.5	Mean 37.0 (SD: 11.4)	100% TLD	6	OT
Skrivankova 2024 [49]	Malawi Zambia	2	Transitioning ART	≤ 400	Malawi: 1422 Zambia: 1410	Malawi: 99 Zambia: 83	Malawi: Median 35 (IQR: 30–40) Zambia: Median 39 (IQR: 33–45)	Malawi: 99.6% TDF + 3TC + DTG or TDF + FTC + DTG; 0.4% ABC + 3TC + DTG Zambia: 85.8% TDF + 3TC + DTG or TDF + FTC + DTG; 14.2% TAF + 3TC + DTG or TAF + FTC + DTG	12; 24	OT
*Retrospective cohort studies*										
Avalos 2019 [36]	Botswana	10	Initiating ART	≤ 400	1,523	61.0	Median 36 (IQR: NR)	100% TDF + FTC + DTG	12; 24	OT
Correa 2020 [34]	Brazil	1	Initiating ART	≤ 50	137	34.3	NR	99.3% TLD; 0.7% ABC + 3TC + DTG	6	OT
Meireles 2019 [38]	Brazil	NR	Initiating ART	≤ 50	11,262	NR	NR	100% TLD	12	ITT
Patel 2021 [60]	India	1	Initiating ART	≤ 1000; ≤ 200; ≤20	86	NR	NR	100% TLD or TAF + FTC + DTG	6; 12	OT
Milward 2023 [61]	Brazil	1	Initiating ART	≤ 1,000; ≤50	47	4.3	Mean 33.9 (SD: 8.1)	100% TLD	6	OT
Silva 2023 [62]	Brazil	NR	Initiating ART	≤ 1000; ≤50	886	19.5	NR	100% TLD	12	OT & ITT
Bareng 2024 [29]	Botswana	NR	Initiating ART	≤ 1000; ≤400; ≤200; ≤50	50,742	59.4	Median 35 (IQR: 29–43)	100% TLD or TDF + FTC + DTG	6; 12; 24; 36; 48; 60	OT
*Cross sectional studies*										
Semengue 2022 [63]	Cameroon	2	Initiating ART; Transitioning ART	≤ 1000; ≤400	310	52.3	Median 41 (IQR: 34–49)	100% TLD	12	OT
Mehari 2021 [35]	Ethiopia	1	Transitioning ART	≤ 50	349	55.0	Mean 40.3 (SD: 11.6)	100% TLD	12	OT
Mahale 2023 [33]	India	1	Initiating ART	≤ 150	70	NR	NR	100% TLD	6; 12	OT

^†^Type of patients: Study participants were reported as people living with HIV initiating ART with a dolutegravir-based ART (initiating ART), patients receiving ART who were transitioned to a dolutegravir-based ART (transitioning ART), or any people living with HIV receiving dolutegravir-based ART. *ABC* abacavir, *ART* antiretroviral therapy, *DTG* dolutegravir, *FTC* emtricitabine, *IQR* interquartile range, *ITT* intention-to-treat, *NR* not reported, *OT* on-treatment, *SD* standard deviation, *TAF* tenofovir alafenamide, *TDF* tenofovir disoproxil fumarate, *TLD* tenofovir disoproxil fumarate + lamivudine + dolutegravir, *3TC* lamivudine

### On-treatment analyses

**Fig. 2:**
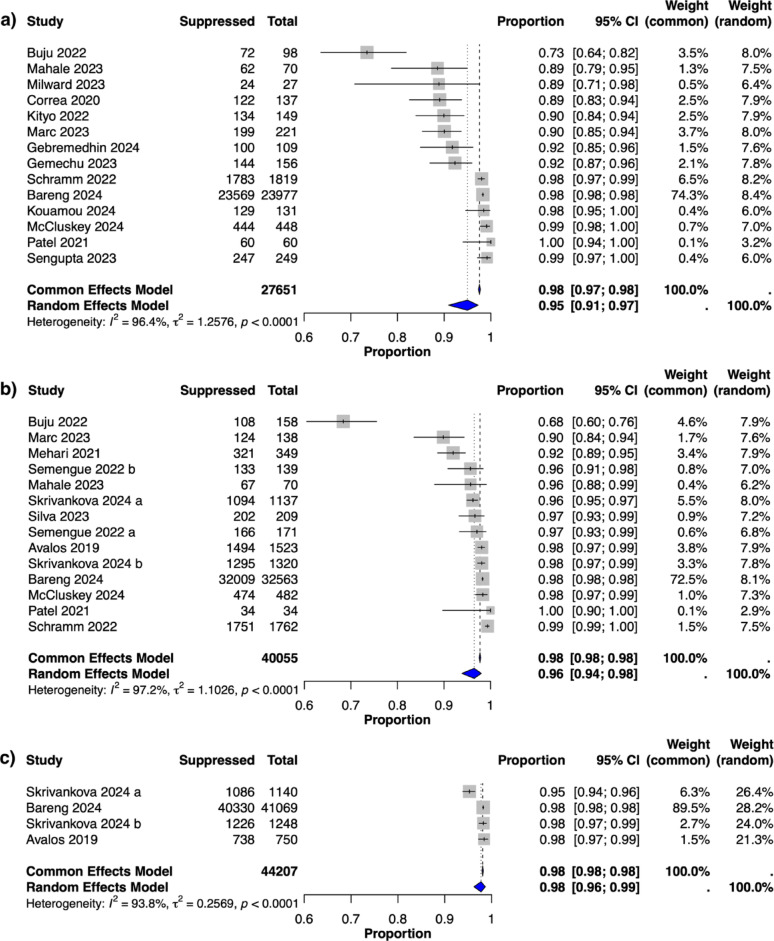
HIV viral suppression among adults receiving WHO-recommended first-line dolutegravir-based ART by treatment duration: On-treatment analysis. Forest plots showing the proportion of HIV viral suppression at (**a**) 6 months, (**b**) 12 months, and (**c**) 24 months among adults receiving WHO-recommended first-line antiretroviral therapy with dolutegravir in programmatic settings. Viral suppression was defined as ≤ 1000 copies/ml or the closest reported threshold, in alignment with WHO standards and UNAIDS monitoring indicators. Each plot displays individual study estimates with their 95% confidence intervals (horizontal lines). The figures show both common-effect and random-effect models for comparison. The blue diamonds represent the pooled prevalence estimates, whereas the grey squares denote the random-effect weights for each study. Two studies are reported as two distinct cohorts because the data were presented separately for each cohort in the original studies: Semengue 2022 is divided into two cohorts: “Semengue 2022 a” represents adults initiating antiretroviral therapy (ART) with a dolutegravir-based regimen, whereas “Semengue 2022 b” represents adults transitioning to dolutegravir-based ART. Similarly, Skrivankova 2024 includes two cohorts: “Skrivankova 2024 a” corresponds to data from Malawi, and “Skrivankova 2024 b” corresponds to data from Zambia

In the on-treatment analysis, the prevalence of viral suppression according to the definition of ≤ 1000 copies/ml was consistently high across the various time points, with random-effects pooled prevalence estimates equal to or exceeding 95% across all time points: 95% (95% CI: 91–97%, I²= 96%) at six months, 96% (95% CI: 94–98%, I²=97%) at 12 months and 98% (95% CI: 96–99%, I²=94%) at 24 months (Fig. 2). Owing to limited data, meta-analyses were not conducted for the ≥ 36-month time points, as only one study was available.

A sensitivity analysis was conducted in which outliers were removed. This resulted in decreased heterogeneity and yielded a slightly lower pooled prevalence estimate at six months but did not affect the pooled estimate at twelve months (Table 2). For the on-treatment analysis at six months, excluding three outlier studies [26, 29, 30] yielded a random-effects pooled viral suppression prevalence of 94% (95% CI: 90–97%, I² = 88%, prediction interval [PI]: 70–99%), down from 95%. At twelve months, the removal of three outliers [26, 29, 31] maintained a pooled prevalence of 96% (95% CI: 95–98%, I²=84%, PI: 87–99%). No outliers were identified at 24 months. Despite the modest change at six months, these results remain consistent overall and support the robustness of the main analysis.

A second sensitivity analysis restricted the dataset to studies that reported ≤ 1000 copies/mL as a viral suppression threshold, excluding those that only used stricter cut-offs (e.g., ≤ 50, ≤150, or ≤ 400 copies/ml). At six months, this viral load threshold-restricted analysis included nine studies and yielded a pooled viral suppression prevalence of 97% (95% CI: 94–99%, I² = 90%, PI: 76–100%), compared with 95% in the main analysis (Table 2). At twelve months, seven studies were included, with a pooled prevalence of 98% (95% CI: 97–99%, I² = 72%, PI: 91–100%), slightly higher than the 96% observed in the main analysis. These findings suggest that excluding studies with stricter viral suppression thresholds may lead to higher pooled viral load suppression prevalence estimates and reduced heterogeneity in meta-analyses.

**Fig. 3:**
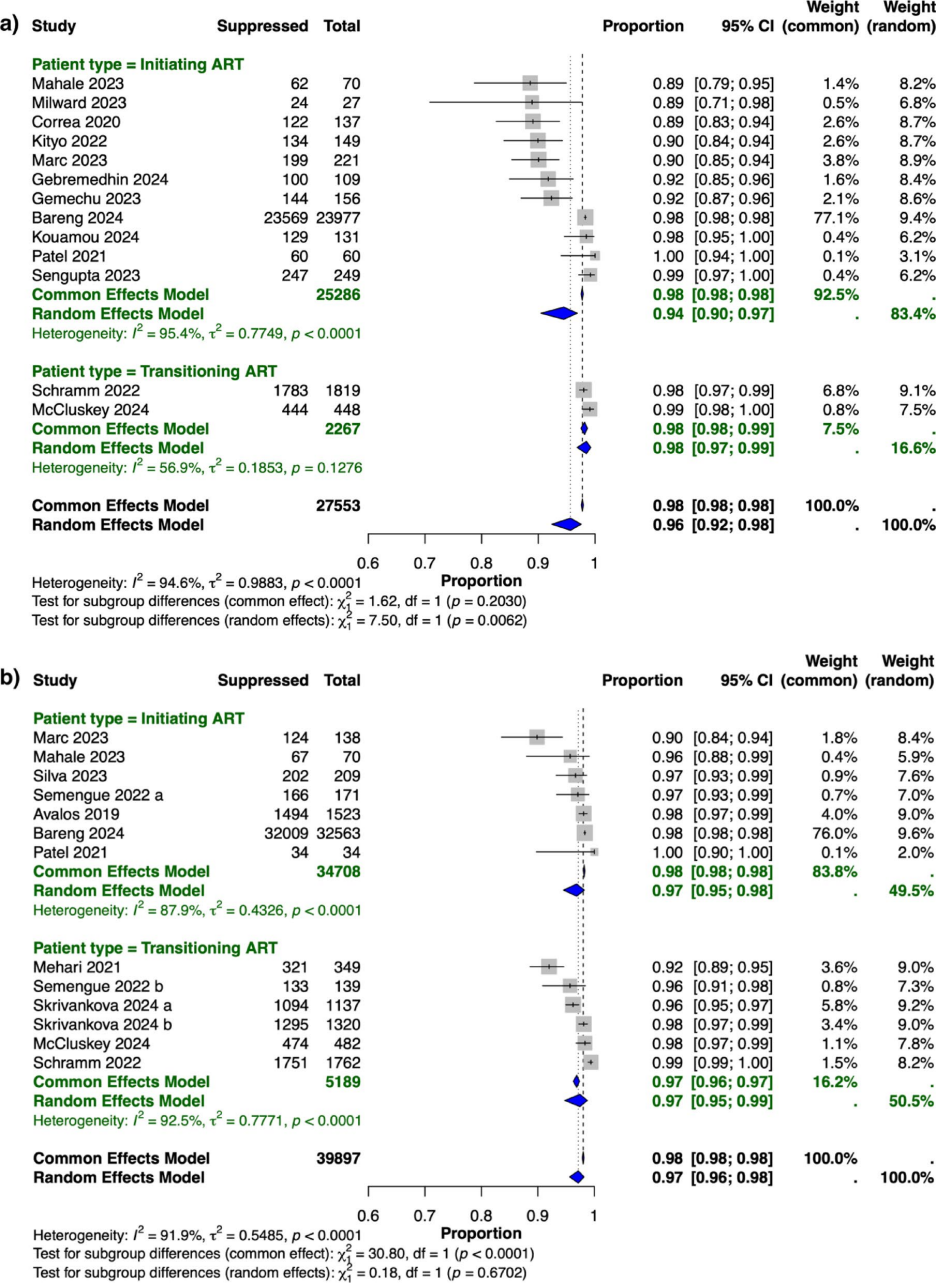
HIV viral suppression among adults receiving WHO-recommended first-line dolutegravir-based ART by patient type and treatment duration: On-treatment analysis. This forest plot displays the results of the subgroup analysis for the prevalence of HIV viral suppression at (**a**) 6 months and (**b**) 12 months among adults receiving WHO-recommended first-line antiretroviral therapy with dolutegravir in programmatic settings. Viral suppression was defined as ≤ 1000 copies/ml or the closest reported threshold, in alignment with WHO standards and UNAIDS monitoring indicators. The analysis is categorised by patient type: Initiating ART (initiating ART with a dolutegravir-based regimen) versus transitioning ART (transitioning to a dolutegravir-based ART). Each plot includes individual study estimates with their 95% confidence intervals (horizontal lines). The figure includes both common-effect and random-effect models for comparison. The blue diamonds represent the pooled prevalence estimates, whereas the grey squares denote the random-effect weights for each study. Two studies are reported as distinct cohorts because the data were presented separately for each cohort in the original studies. Semengue 2022 is divided into two cohorts: “Semengue 2022a” represents adults initiating antiretroviral therapy (ART) with a dolutegravir-based regimen, whereas “Semengue 2022b” represents adults transitioning to dolutegravir-based ART. Similarly, Skrivankova 2024 includes two cohorts: “Skrivankova 2024a” corresponds to data from Malawi, and “Skrivankova 2024 b” corresponds to data from Zambia

Subgroup analyses were conducted for initiating versus transitioning to a dolutegravir-based regimen using 6-month and 12-month viral suppression data; however, the 24-month analysis was not performed because of the limited number of studies (*n* = 4). At six months, eleven studies contributed data for the initiating group and two for the transitioning group. In the on-treatment subgroup analysis, the random-effects pooled prevalence of viral suppression at six months was 94% (95% CI: 90–97%, I² = 95%) for patients initiating ART with a dolutegravir-based regimen and 98% (95% CI: 97–99%, I²= 57%, *p* < 0.01) for patients transitioning to dolutegravir-based ART regimens (Fig. 3a). The significant difference between subgroups at six months may reflect the fact that many individuals who transitioned were already virally suppressed and maintained suppression following the regimen change. At 12 months, seven studied groups contributed data for the initiating group and six for the transitioning group. The pooled prevalence was 97% (95% CI: 95–98%, I²=88%) for those initiating ART with a dolutegravir-containing regimen and 97% (95% CI: 95–99%, I² = 93%, *p* = 0.67) for those transitioning to a dolutegravir-based regimen (Fig. 3b), suggesting that both patient groups exhibited similarly high prevalences of viral suppression after one year of treatment.

### Intention-to-treat analyses

**Fig. 4:**
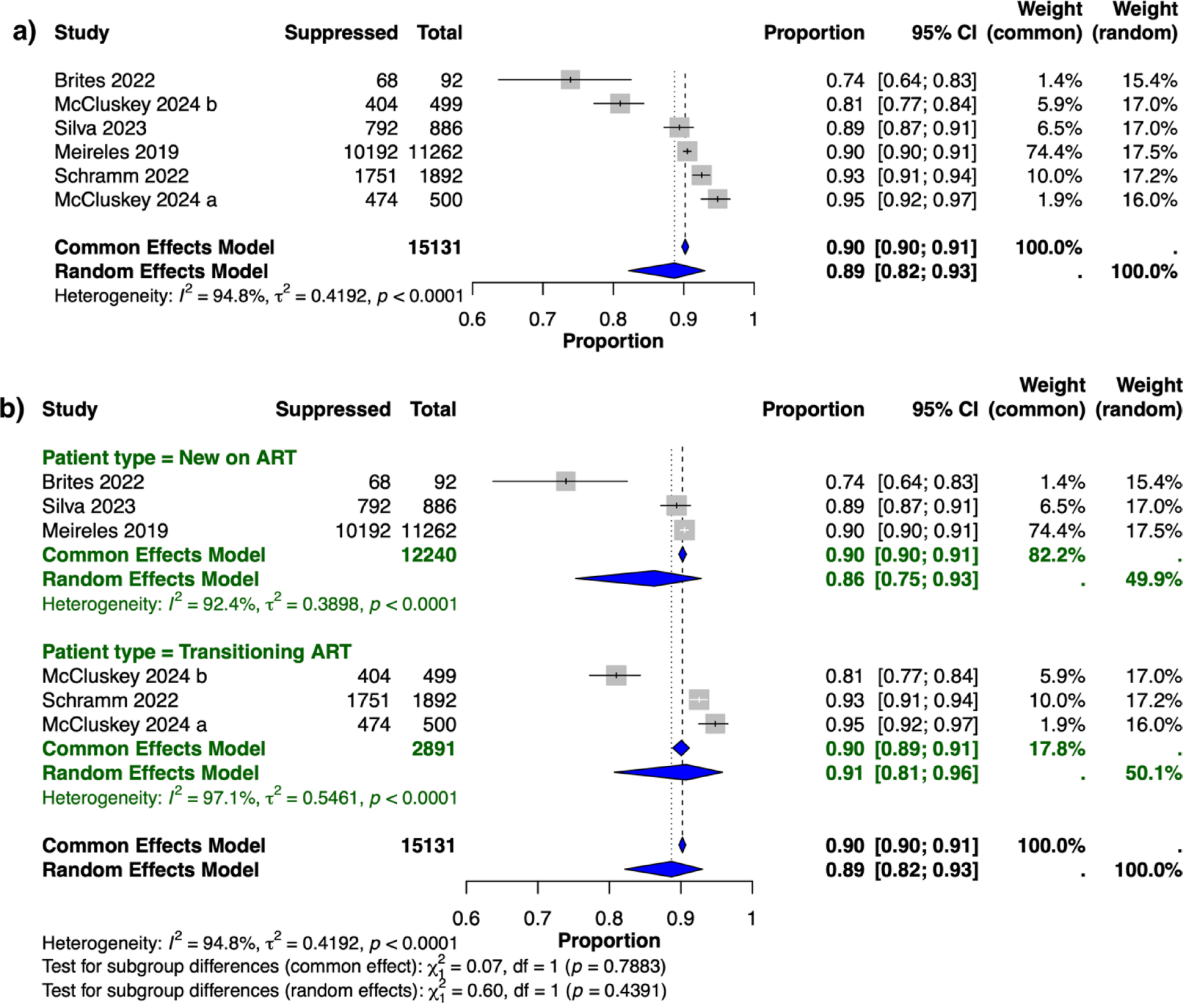
HIV viral suppression among adults receiving WHO-recommended first-line dolutegravir-based ART: Intention-to-treat analysis. (**a**) Forest plot showing the overall proportion of adults who achieved HIV viral suppression (≤ 1000 copies/ml) at 12 months after initiating WHO-recommended first-line dolutegravir-based antiretroviral therapy in programmatic settings. (**b**) Forest plot of the same 12-month HIV viral suppression outcome, stratified by patient type (“New on ART” vs. “Transitioning ART”). Viral suppression was defined as ≤ 1000 copies/ml or the closest reported threshold, in alignment with WHO standards and UNAIDS monitoring indicators. Each study’s estimate is represented by a grey square (sized according to its weight under the random-effects model), with horizontal lines indicating 95% confidence intervals. Both common-effect and random-effects summary estimates are displayed, with the pooled effect represented by a blue diamond. McCluskey 2024 a and McCluskey 2024 b refer to two distinct studies by the same first author published in the same year, conducted in Uganda and South Africa, respectively. Both analyses follow an intention-to-treat approach

Under the intention-to-treat analysis, the random-effects pooled prevalence of 12-month viral suppression was 89% (95% CI: 82–93%, I² = 95%) (Fig. 4a). When stratified by patient type (Fig. 4b), the random-effects pooled prevalence was 86% (95% CI: 75–93%) among those newly initiating dolutegravir-based ART and 91% (95% CI: 81–96%) among those transitioning from a non-dolutegravir regimen. Tests for subgroup differences were not significant (*p* = 0.44 for random-effects), indicating that both groups achieved similarly high rates of 12-month viral suppression.

A sensitivity analysis was conducted for the 12-month intention-to-treat outcome by removing an outlier study [28], which modestly increased the pooled prevalence to 90% (95% CI: 86–94%) and slightly reduced heterogeneity (I² = 94) (Table 2). These findings remained consistent with the main analysis and support the robustness of the intention-to-treat estimates.

Due to limited data availability at other time points, meta-analyses under the intention-to-treat approach were conducted only for the 12-month outcome. Overall, these results mirrored the robust viral suppression observed in the on-treatment analyses, albeit with slightly lower pooled estimates, which were consistent with the more conservative assumption of the intention-to-treat analyses.

### Quality and certainty of evidence

Regarding the risk of bias (Supplementary Material 4), cohort studies generally had valid outcome measurements, adequate follow-up, and appropriate statistical analyses. While issues with confounding factors and incomplete follow-up were identified, these were not considered critical sources of bias, given the focus of this review on prevalence estimates rather than measures of association. Cross-sectional studies had suitable sampling frames and valid methods for measuring the condition and conducting statistical analyses. However, two of the three studies had unclear or inadequate sampling methods and sample sizes, which could impact their generalizability. Despite the described methodological limitations, the included studies were considered applicable to the target population, and the pooled prevalence estimates demonstrated acceptable precision. High heterogeneity was observed across multiple time periods, but sensitivity and subgroup analyses helped reduce some variability and provided additional context for the findings.

The certainty of the evidence for 12-month viral suppression outcomes was rated as moderate for both the on-treatment and intention-to-treat analyses, based on the GRADE assessment (Supplementary Material 5). For the 12-month on-treatment analysis, which included 14 studies and over 40,000 participants, the certainty was downgraded due to inconsistency (I² = 97%). However, the large sample size and narrow confidence interval (96%; 95% CI: 94–98%) support a robust pooled estimate. For the 12-month intention-to-treat analysis, which included six studies and over 15,000 participants, the certainty was downgraded for both inconsistency (I² = 95%) and imprecision, due to the wider confidence interval (89%; 95% CI: 82–93%) and a smaller number of contributing studies. In both cases, the studies were deemed applicable to the review population and produced meaningful, programmatically relevant estimates.

**Table 2:** Sensitivity analysis of HIV viral suppression prevalence among adults receiving WHO-recommended first-line dolutegravir-based ART

Analysis	Number of cohorts studied	Number of participants with viral suppression	Number of participants	Pooled viral suppression prevalence ^¶^		I² statistic		Prediction interval
				Prevalence	95% CI	I²	95% CI	
*On-treatment analysis*								
6 Months^†^								
Main analysis	14	27,089	27,651	95%	91–97%	96%	95–97%	60–100%
Outlier removed	11	3004	3128	94%	90–97%	88%	80–93%	70–99%
Viral load threshold restricted	9	26,534	27,016	97%	94–99%	90%	84–94%	76–100%
12 Months^‡^								
Main analysis	14	39,272	40,055	96%	94–98%	97%	96–98%	72–100%
Outlier removed	11	5404	5,572	96%	95–98%	84%	73–91%	87–99%
Viral load threshold restricted	7	34,769	35,360	98%	97–99%	72%	39–87%	91–100%
*Intention-to-treat analysis*								
12 Months^§^								
Main analysis	6	13,681	15,131	89%	82–93%	95%	91–97%	56–98%
Outlier removed	5	13,613	15,039	90%	86–94%	94%	90–97%	66–98%
Viral load threshold restricted	3	3017	3278	92%	89–95%	86%	58–95%	68–99%

^†^6-month on-treatment analyses: Buju 2022, Bareng 2024, and McCluskey 2024 [26, 29, 30] were removed as outliers. Gebremedhin 2024, Mahale 2023, Buju 2022, Correa 2020, and Marc 2023 [26, 27, 32–34] were removed from the viral load threshold–restricted analysis for using stricter thresholds (< 1000 copies/ml)

^‡^12-month on-treatment analyses: Buju 2022, Schramm 2022, and Bareng 2024 [26, 29, 31] were removed as outliers. Mahale 2023, Mehari 2021, Buju 2022, Marc 2023, Avalos 2019, and Skrivankova 2024 (a and b) were not included in the viral load threshold–restricted analysis [26, 27, 33, 35–37]

^§ ^12-month intention-to-treat analyses: Brites 2022 [28] was removed as an outlier. Meireles 2019, Brites 2022, and McCluskey 2024 b [28, 38, 39] were not included in the viral load threshold–restricted analysis

^¶^All pooled prevalence estimates were calculated using a random-effects model. Viral suppression was defined as ≤ 1000 copies/ml or the closest reported threshold, in alignment with WHO standards and UNAIDS monitoring indicators

## Discussion

This is the first systematic review and meta-analysis to assess the prevalence of HIV viral suppression among adults receiving WHO-recommended first-line dolutegravir-based ART regimens [5] in programmatic settings across LMICs, showing that these regimens achieve a high prevalence of viral suppression across multiple time points. The on-treatment analysis revealed high pooled prevalence rates of viral suppression at six months (95%), 12 months (96%), and 24 months (98%). These findings align with the global HIV treatment goals set by UNAIDS, which aim to achieve 95% viral suppression among people receiving ART by 2030 [10]. The observed high prevalence of viral suppression suggests that dolutegravir-based ART regimens can be highly effective among adults retained in care in programmatic settings. These findings are particularly relevant as maintaining high levels of viral suppression is crucial for reducing HIV transmission and improving health outcomes among people living with HIV receiving ART [21].

The intention-to-treat analysis revealed a slightly lower but still high pooled viral suppression prevalence at 12 months (89%). The high prevalence of viral suppression observed in the on-treatment analysis indicates that patients who remain on dolutegravir-based ART are likely to achieve viral suppression, whereas the intention-to-treat findings provide a more realistic estimate of viral suppression prevalence in real-world settings, where HIV treatment interruptions and loss-to-follow-up are common challenges [12, 13]. These results underscore the need for strategies to enhance ART adherence and retention in care to maximise the benefits of dolutegravir-based ART regimens.

Notable improvements in virological outcomes in LMICs have been observed with the introduction of WHO-recommended dolutegravir-based ART regimens compared with earlier research by Boender et al. [40], who reported on-treatment virological suppression rates of 84.9% at six months, 85.6% at 12 months, and 84.4% at 24 months among adults receiving first-line ART in the pre-dolutegravir era. These rates are lower than those reported in this systematic review focusing on dolutegravir-based regimens, suggesting that the enhanced potency, tolerability, and high genetic barrier to resistance of dolutegravir-based ART [41–43] may have contributed to the higher rates of viral suppression recorded across various time points. Although not examined in this review, advancements in HIV care over time may also have contributed to these improved outcomes.

Moreover, in this systematic review, the intention-to-treat analysis for WHO-recommended dolutegravir-based regimens revealed a viral suppression rate of 89% at 12 months, which is considerably higher than the 67.3% reported by Boender et al. [40]. While this difference may suggest that dolutegravir-based regimens are associated with higher patient retention and better virological outcomes even when treatment interruptions and loss to follow-up occur, it is also likely that enhanced retention strategies, such as differentiated service delivery, close follow-up for patients at risk of being lost to follow-up, and the integration of HIV services with other health services, have contributed to this effect [44–46]. Kanters et al. [41], in a systematic review and network meta-analysis of randomised clinical trials, reported that dolutegravir-based ART tends to be protective against ART discontinuation due to adverse events compared with normal-dose efavirenz, the regimen most commonly used in studies reported by Boender et al. [40]. This protective effect, combined with improved retention strategies, suggests that dolutegravir-based ART may offer significant advantages in maintaining treatment continuity, which is essential for achieving and sustaining viral suppression.

Notable differences in study numbers and geographical representation are evident between the current systematic review and that conducted by Boender et al. [40]. While this systematic review analysed data from 22 studies across 13 countries, with significant contributions from Brazil, Ethiopia, India, and Malawi, Boender et al.‘s analysis included 163 studies across 35 countries, predominantly from sub-Saharan Africa. The smaller sample size and narrower geographical coverage in this review may limit the generalizability of the findings and precluded meaningful stratified analysis by WHO region or income group. Given these differences, it is advisable to repeat the current analyses as more data from a broader range of LMICs become available over time. Reevaluating the findings with additional studies from diverse geographical regions could provide deeper insights into the regional differences in virological suppression.

The sensitivity analyses conducted to address the initial high heterogeneity effectively reduced variability and reinforced the reliability of the viral suppression outcomes. When outliers were excluded, heterogeneity at six months decreased from 96 to 88%, lowering the pooled prevalence from 95 to 94%, although it remained within the original 95% CI. At 12 months, while the pooled prevalence remained at 96%, heterogeneity also decreased from 97 to 84%. These findings suggest that the pooled outcomes are robust and not unduly influenced by extreme values, supporting the effectiveness of WHO-recommended first-line dolutegravir-based ART for adults in LMIC settings.

A second sensitivity analysis restricted the dataset to studies that reported ≤ 1,000 copies/ml as a viral suppression threshold, excluding those that only used stricter definitions (e.g., ≤ 50, ≤150, or ≤ 400 copies/ml). This restriction yielded slightly higher pooled viral suppression prevalence and reduced heterogeneity at both six and twelve months. These findings suggest that the use of stricter viral suppression thresholds may lower prevalence estimates by classifying more individuals as unsuppressed, even when viral levels are clinically low and programmatically acceptable. Such variation in outcome definitions introduces measurement inconsistency across studies and can contribute to statistical heterogeneity in meta-analyses. These results underscore the importance of harmonised viral suppression definitions—aligned with WHO and UNAIDS standards—when reporting programmatic data to ensure comparability and reliability of pooled estimates.

However, it is important to acknowledge that some residual heterogeneity remained even after outlier removal and threshold restriction. This could be attributed to unmeasured factors such as differences in patient demographics, healthcare infrastructure, the duration of prior ART regimens, and socioeconomic conditions across the included studies [47]. Although exploring these potential sources in depth was not feasible, a descriptive overview provides further context: most studies were conducted in sub-Saharan Africa, with others from Asia and Latin America; study designs included prospective and retrospective cohorts as well as cross-sectional studies; and both individuals initiating ART and those transitioning from other regimens were represented. While treatment status (ART initiation versus transition) was examined through subgroup analysis, other relevant sources of heterogeneity, such as geographic setting, methodological approach, and participant characteristics, could not be formally assessed due to data limitations. Expanding regional and methodological representation in future studies would not only improve generalizability but also enable more granular subgroup analyses to better understand heterogeneity across diverse programmatic contexts.

The subgroup analyses provided valuable insights into the dynamics of treatment initiation versus transition with dolutegravir-based ART regimens. At six months, the random-effects model for the on-treatment analysis revealed a significant difference between patients initiating ART with dolutegravir-based regimens and those transitioning from other regimens, with the latter group exhibiting slightly higher viral suppression rates (94% vs. 98%, *p* < 0.01). This difference might be explained by viral load decay, as patients initiating treatment may start with much higher viral loads, whereas those transitioning are more likely to have already achieved viral suppression or have lower baseline viral loads. By twelve months, however, these differences disappeared, with both individuals initiating ART and those transitioning from other regimens exhibiting a pooled prevalence of viral suppression of 97%. This convergence indicates that the effectiveness of dolutegravir-based ART stabilises across different patient populations within a year. These findings reinforce the regimen’s sustained effectiveness and broad applicability, regardless of whether patients are initiating treatment or transitioning from previous ART.

While the absence of viral load data should not prevent transitioning to dolutegravir-based ART [5], it remains critical to assess whether viral suppression and drug resistance outcomes differ based on viral load status during transition [48]. A limitation of this review is that only five studies reported viral suppression outcomes among adults transitioning to dolutegravir-based ART, with only two (Schramm et al. and Skrivankov et al. [31, 49]) providing disaggregated analysis by viral load status at the time of ART transition. Schramm et al. reported that a high baseline viral load was associated with viral failure, with an adjusted odds ratio of 14.1 (95% CI: 2.3–87.4) for viral loads between 1000 and < 10,000 copies/ml and an adjusted odds ratio of 64.4 (95% CI: 19.3–215.4) for viral loads ≥ 10,000 copies/ml [31]. Similarly, Skrivankov et al. reported a significantly higher likelihood of remaining viremic at one and two years post-transition among individuals who switched with a viral load ≥ 1,00 copies/ml, reporting adjusted odds ratios of 7.10 (95% CI: 3.17–14.63) and 11.92 (95% CI: 5.97–22.87), respectively, than those who were virologically suppressed at the time of transition [49]. Additionally, a study in Mozambique reported increased HIV drug resistance to dolutegravir among individuals who transitioned with unsuppressed or unknown viral loads [50], with resistance rates of 11.4% for those with a suppressed viral load, 17.8% for those with an unsuppressed viral load, and 37.5% for those with an unknown viral load status. Given that the transition to dolutegravir-based ART has been widely implemented in many countries, these findings highlight the need for enhanced adherence counselling and virologic monitoring for individuals who have transitioned without viral suppression. This may be particularly important, as such individuals may not receive the same level of adherence support typically provided when switching to second-line therapy, despite being on a similar regimen.

In addition to the limitations already described, this systematic review and meta-analysis has several other constraints. First, the long-term effectiveness of dolutegravir-based ART was not evaluated because of the limited availability of data beyond 24 months in the on-treatment analysis. This gap prevents a comprehensive understanding of the regimen’s sustained effectiveness over extended periods. Given that WHO-recommended dolutegravir-based ART was introduced in the last few years, more long-term data are likely yet to be generated and published. Future studies from programmatic settings will be essential as more data become available to complement the analysis and better understand the long-term effectiveness of WHO-recommended dolutegravir-based ART. Second, few studies have reported intention-to-treat outcomes, which limits the ability to assess the full impact of treatment interruptions and loss-to-follow-up on viral suppression rates. Third, although the viral load monitoring capacity is expanding in LMICs, substantial gaps remain. A recent systematic review reported wide variability in initial viral load monitoring coverage across study settings, ranging from 12 to 93%, with a median of 74% (IQR: 46–82%) [51]. Coverage was often higher in settings with external donor support or innovative models of care but significantly lower in decentralised facilities without such support—for example, viral load coverage ranged from 25 to 81% in South Africa depending on the site [57]. These disparities mean that estimates of viral suppression derived from programmatic data may not fully represent the broader population, particularly in under-resourced areas where testing access is limited. Fourth, approximately one-fifth of included studies were identified from conference abstracts. While abstracts may sometimes lack methodological detail, only one of the five abstracts included in this review had insufficient information to assess one risk-of-bias criterion (“Were confounding factors identified?”), which was rated as “unclear”. Finally, poor outcomes may have been underreported, which could lead to an overestimation of the regimen’s effectiveness in real-world settings.

While this study has limitations, it has significant strengths that underscore its contribution to understanding the clinical outcomes of WHO-recommended dolutegravir-based ART in LMICs. First, the study synthesises data from real-world settings, offering a comprehensive assessment of viral suppression outcomes in environments facing resource constraints. Second, including on-treatment and intention-to-treat analyses allows for a more nuanced understanding of the effectiveness of dolutegravir-based regimens, considering both optimal and more challenging clinical scenarios. Third, the sensitivity analyses have reinforced the findings’ robustness, ensuring that the pooled prevalence estimates are reliable and not overly influenced by extreme cases. Finally, the subgroup analyses add a layer of insight by examining differences in viral suppression between patients initiating dolutegravir-based ART and those transitioning to dolutegravir-based regimens, offering valuable information on these two treatment strategies recommended by WHO.

This systematic review and meta-analysis show that WHO-recommended first-line dolutegravir-based ART regimens achieve very high viral suppression among adults in LMICs—even out to two years—thereby highlighting their pivotal contribution toward global HIV treatment objectives, including the UNAIDS target of 95% suppression by 2030. Sustained viral suppression not only improves individual health outcomes but also plays a critical role in preventing onward transmission and reducing HIV-related mortality. However, ensuring continued viral suppression at scale relies on continuous access to treatment, an assurance now under threat. Recent international funding cuts threaten to erode these hard-won advances. One modelling exercise, drawing on 26 country-validated Optima HIV models, projected that a 24% drop in international HIV assistance during 2025–26 could produce an additional 4.4–10.8 million new infections and 0.8–2.9 million HIV-related deaths in LMICs by 2030 if financing is not restored [52]. In seven high-burden sub-Saharan African countries, an STDSIM simulation estimated 60,000 excess deaths (95% UI 49,000–71,000) under a proportional-loss scenario, increasing to 74,000 deaths (95% UI 63,000–89,000), assuming near-total system collapse, and 35,000–103,000 extra infections over the same period [53]. Together, these projections underscore that, despite the proven effectiveness of dolutegravir-based regimens at the individual level, safeguarding population-level viral suppression will require stable, diversified funding and resilient delivery systems to prevent a backslide in epidemic control.

## Conclusion

This systematic review and meta-analysis suggests that WHO-recommended first-line dolutegravir-based ART regimens are highly effective in achieving viral suppression among adults in LMICs, with high levels of viral suppression observed even up to two years. These findings underscore the important role of WHO-recommended dolutegravir-based ART in helping achieve global HIV treatment targets, including the UNAIDS goal of 95% viral suppression by 2030. However, the study also highlights the need for ongoing research to assess long-term effectiveness and address gaps in viral load monitoring and reporting.

## Supplementary Information

**Supplementary Material 1. Appendix S1.** The PRISMA checklist for abstracts. **Appendix S2.** The PRISMA checklist. **Appendix S3.** Search strategy used in each database. **Appendix S4.** Risk of Bias Assessment for Included Studies. **Appendix S5.** GRADE Assessment.

## Data Availability

The data that support the findings of this study are available from the corresponding author upon reasonable request.

## Abbreviations

ART: Antiretroviral therapy
ARV: Antiretroviral
CI: Confidence interval
CROI: Conference on Retroviruses and Opportunistic Infections
DTG: Dolutegravir
IAS: International AIDS Society
I²: Heterogeneity statistic
ITT: Intention-to-treat
LMIC(s): Low- and middle-income country(ies)
OT: On-treatment
PI: Prediction interval
PRISMA: Preferred Reporting Items for Systematic Reviews and Meta-Analyses
PROSPERO: International Prospective Register of Systematic Reviews
UNAIDS: Joint United Nations Programme on HIV/AIDS
VL: Viral load
WHO: World Health Organization
