# Supplementary material for: High HIV viral suppression among adults receiving WHO-recommended first-line dolutegravir-based antiretroviral therapy in low- and middle-income countries: a systematic review and meta-analysis of programmatic evidence
Source: AIDS Res Ther. 2025 Sep 26;22:91. doi: 10.1186/s12981-025-00788-8 (PMC12465354; doi:10.1186/s12981-025-00788-8)
Supplement: Supplementary file 1 — Supplementary Material 1. Appendix S1. The PRISMA checklist for abstracts. Appendix S2. The PRISMA checklist. Appendix S3. Search strategy used in each database. Appendix S4. Risk of Bias Assessment for Included Studies. Appendix S5. GRADE Assessment. [file 12981_2025_788_MOESM1_ESM.docx]

**SUPPLEMENTARY INFORMATION**

High HIV Viral Suppression among Adults Receiving WHO-Recommended First-Line Dolutegravir-Based Antiretroviral Therapy in Low- and Middle-Income Countries: A Systematic Review and Meta-Analysis of Programmatic Evidence

Table of Contents

[Appendix S1. The PRISMA checklist for abstracts 2](#_Toc204628867)

[Appendix S2. The PRISMA checklist 3](#_Toc204628868)

[Appendix S3. Search strategy used in each database 6](#_Toc204628869)

[Appendix S4. Risk of Bias Assessment for Included Studies 10](#_Toc204628870)

[Appendix S5. GRADE Assessment 12](#_Toc204628871)

# **Appendix S1. The PRISMA checklist for abstracts**

| **Section and Topic** | **Item #** | **Checklist item** | **Reported (Yes/No)** |
| --- | --- | --- | --- |
| **TITLE** | | |  |
| Title | 1 | Identify the report as a systematic review. | Yes |
| **BACKGROUND** | | |  |
| Objectives | 2 | Provide an explicit statement of the main objective(s) or question(s) the review addresses. | Yes |
| **METHODS** | | |  |
| Eligibility criteria | 3 | Specify the inclusion and exclusion criteria for the review. | Yes |
| Information sources | 4 | Specify the information sources (e.g. databases, registers) used to identify studies and the date when each was last searched. | Yes |
| Risk of bias | 5 | Specify the methods used to assess risk of bias in the included studies. | Yes |
| Synthesis of results | 6 | Specify the methods used to present and synthesise results. | Yes |
| **RESULTS** | | |  |
| Included studies | 7 | Give the total number of included studies and participants and summarise relevant characteristics of studies. | Yes |
| Synthesis of results | 8 | Present results for main outcomes, preferably indicating the number of included studies and participants for each. If meta-analysis was done, report the summary estimate and confidence/credible interval. If comparing groups, indicate the direction of the effect (i.e. which group is favoured). | Yes |
| **DISCUSSION** | | |  |
| Limitations of evidence | 9 | Provide a brief summary of the limitations of the evidence included in the review (e.g. study risk of bias, inconsistency and imprecision). | Yes (Results) |
| Interpretation | 10 | Provide a general interpretation of the results and important implications. | Yes (Conclusion) |
| **OTHER** | | |  |
| Funding | 11 | Specify the primary source of funding for the review. | Yes (on the main paper) |
| Registration | 12 | Provide the register name and registration number. | Yes (on the main paper) |

# **Appendix S2. The PRISMA checklist**

| **Section and Topic** | **Item #** | **Checklist item** | **Location where item is reported** |
| --- | --- | --- | --- |
| **TITLE** | | |  |
| Title | 1 | Identify the report as a systematic review. | At the end of the title |
| **ABSTRACT** | | |  |
| Abstract | 2 | See the PRISMA 2020 for Abstracts checklist. | Abstract and Appendix S1. The PRIMSA checklist for abstracts |
| **INTRODUCTION** | | |  |
| Rationale | 3 | Describe the rationale for the review in the context of existing knowledge. | Introduction: 3^rd^ paragraph |
| Objectives | 4 | Provide an explicit statement of the objective(s) or question(s) the review addresses. | Introduction: 4^th^ paragraph |
| **METHODS** | | |  |
| Eligibility criteria | 5 | Specify the inclusion and exclusion criteria for the review and how studies were grouped for the syntheses. | Methods: Search Strategy and Study Selection, 3^rd^ and 4^th^ paragraphs. |
| Information sources | 6 | Specify all databases, registers, websites, organisations, reference lists and other sources searched or consulted to identify studies. Specify the date when each source was last searched or consulted. | Methods: Search Strategy and Study Selection, 1^st^ paragraph. |
| Search strategy | 7 | Present the full search strategies for all databases, registers and websites, including any filters and limits used. | Appendix S3 |
| Selection process | 8 | Specify the methods used to decide whether a study met the inclusion criteria of the review, including how many reviewers screened each record and each report retrieved, whether they worked independently, and if applicable, details of automation tools used in the process. | Methods: Search Strategy and Study Selection, 2^nd^ paragraph. |
| Data collection process | 9 | Specify the methods used to collect data from reports, including how many reviewers collected data from each report, whether they worked independently, any processes for obtaining or confirming data from study investigators, and if applicable, details of automation tools used in the process. | Methods: Data Extraction |
| Data items | 10a | List and define all outcomes for which data were sought. Specify whether all results that were compatible with each outcome domain in each study were sought (e.g. for all measures, time points, analyses), and if not, the methods used to decide which results to collect. | Methods: Data Extraction |
|  | 10b | List and define all other variables for which data were sought (e.g. participant and intervention characteristics, funding sources). Describe any assumptions made about any missing or unclear information. | Methods: Data Extraction |
| Study risk of bias assessment | 11 | Specify the methods used to assess risk of bias in the included studies, including details of the tool(s) used, how many reviewers assessed each study and whether they worked independently, and if applicable, details of automation tools used in the process. | Methods: Certainty of Evidence |
| Effect measures | 12 | Specify for each outcome the effect measure(s) (e.g. risk ratio, mean difference) used in the synthesis or presentation of results. | Methods: Statistical Analysis, 1^st^ paragraph |
| Synthesis methods | 13a | Describe the processes used to decide which studies were eligible for each synthesis (e.g. tabulating the study intervention characteristics and comparing against the planned groups for each synthesis (item #5)). | Methods: Statistical Analysis, 2^nd^ paragraph |
|  | 13b | Describe any methods required to prepare the data for presentation or synthesis, such as handling of missing summary statistics, or data conversions. | Methods: Statistical Analysis, 2^nd^ paragraph |
|  | 13c | Describe any methods used to tabulate or visually display results of individual studies and syntheses. | Methods: Statistical Analysis, 1^st^ paragraph |
|  | 13d | Describe any methods used to synthesize results and provide a rationale for the choice(s). If meta-analysis was performed, describe the model(s), method(s) to identify the presence and extent of statistical heterogeneity, and software package(s) used. | Methods: Statistical Analysis, 1^st^ and 3^rd^ paragraphs |
|  | 13e | Describe any methods used to explore possible causes of heterogeneity among study results (e.g. subgroup analysis, meta-regression). | Methods: Statistical Analysis, 3^rd^ paragraph |
|  | 13f | Describe any sensitivity analyses conducted to assess robustness of the synthesized results. | Methods: Statistical Analysis, 3^rd^ paragraph |
| Reporting bias assessment | 14 | Describe any methods used to assess risk of bias due to missing results in a synthesis (arising from reporting biases). | Methods: Certainty of Evidence |
| Certainty assessment | 15 | Describe any methods used to assess certainty (or confidence) in the body of evidence for an outcome. | Methods: Certainty of Evidence |
| **RESULTS** | | |  |
| Study selection | 16a | Describe the results of the search and selection process, from the number of records identified in the search to the number of studies included in the review, ideally using a flow diagram. | Results: 1^st^ paragraph and Figure 1 |
|  | 16b | Cite studies that might appear to meet the inclusion criteria, but which were excluded, and explain why they were excluded. | None |
| Study characteristics | 17 | Cite each included study and present its characteristics. | Results: 1^st^ paragraph and Table 1 |
| Risk of bias in studies | 18 | Present assessments of risk of bias for each included study. | Results: 6^th^ paragraph and Supplementary Material 4 |
| Results of individual studies | 19 | For all outcomes, present, for each study: (a) summary statistics for each group (where appropriate) and (b) an effect estimate and its precision (e.g. confidence/credible interval), ideally using structured tables or plots. | Results: 1^st^ paragraph, Table 1 and Figures 2 and 3 |
| Results of syntheses | 20a | For each synthesis, briefly summarise the characteristics and risk of bias among contributing studies. | Results: 2^nd^ and 5^th^ paragraphs |
|  | 20b | Present results of all statistical syntheses conducted. If meta-analysis was done, present for each the summary estimate and its precision (e.g. confidence/credible interval) and measures of statistical heterogeneity. If comparing groups, describe the direction of the effect. | Results: 2^nd^ and 5^th^ paragraphs and Figures 2 and 4 |
|  | 20c | Present results of all investigations of possible causes of heterogeneity among study results. | Results: 3^rd^, 4^th^, and 5^th^ paragraphs, Table 2 and Figures 3 and 4 |
|  | 20d | Present results of all sensitivity analyses conducted to assess the robustness of the synthesized results. | Results: 3^rd^ paragraph and Table 2 |
| Reporting biases | 21 | Present assessments of risk of bias due to missing results (arising from reporting biases) for each synthesis assessed. | Results: 6^th^ paragraph and Supplementary Material 4 |
| Certainty of evidence | 22 | Present assessments of certainty (or confidence) in the body of evidence for each outcome assessed. | Results: 6^th^ paragraph and Supplementary Material 4 |
| **DISCUSSION** | | |  |
| Discussion | 23a | Provide a general interpretation of the results in the context of other evidence. | Discussion: paragraphs 1-9 |
|  | 23b | Discuss any limitations of the evidence included in the review. | Discussion: paragraph 10 |
|  | 23c | Discuss any limitations of the review processes used. | Discussion: paragraph 10 |
|  | 23d | Discuss implications of the results for practice, policy, and future research. | Discussion: paragraphs 1-9 |
| **OTHER INFORMATION** | | |  |
| Registration and protocol | 24a | Provide registration information for the review, including register name and registration number, or state that the review was not registered. | Methods: 1^st^ paragraph |
|  | 24b | Indicate where the review protocol can be accessed, or state that a protocol was not prepared. | Methods: 1^st^ paragraph |
|  | 24c | Describe and explain any amendments to information provided at registration or in the protocol. | Not applicable |
| Support | 25 | Describe sources of financial or non-financial support for the review, and the role of the funders or sponsors in the review. | Funding |
| Competing interests | 26 | Declare any competing interests of review authors. | Competing interests |
| Availability of data, code and other materials | 27 | Report which of the following are publicly available and where they can be found: template data collection forms; data extracted from included studies; data used for all analyses; analytic code; any other materials used in the review. | Data Availability Statement |

# **Appendix S3. Search strategy used in each database**

Ovid MEDLINE(R) ALL <1946 to September 26, 2024>

| **Step** | **Query** | **Results** |
| --- | --- | --- |
| 1 | exp HIV Infections/ | 325479 |
| 2 | exp HIV/ | 109711 |
| 3 | exp Acquired Immunodeficiency Syndrome/ | 79388 |
| 4 | (HIV or HIV-1 or human immunodeficiency virus or human immuno deficiency virus or human immunedeficiency virus or human immune deficiency virus or AIDS or acquired immunodeficiency syndrome or acquired immuno deficiency syndrome or acquired immunedeficiency syndrome or acquired immune deficiency syndrome).tw. | 478524 |
| 5 | (HIV or HIV-1 or human immunodeficiency virus or human immuno deficiency virus or human immune deficiency virus).tw. | 386805 |
| 6 | (AIDS or acquired immunodeficiency syndrome or acquired immuno deficiency syndrome or acquired immune deficiency syndrome).tw. | 176951 |
| 7 | 1 or 2 or 3 or 4 or 5 or 6 | 517002 |
| 8 | (dolutegravir* or DTG* or TLD* or Tivicay or JUL or Juluca or dovato or triumeq or soltegravir or GSK1349572 or "S/GSK1349572" or "GSK-1349572" or "GSK 1349572").tw. | 9513 |
| 9 | exp Viral Load/ | 40144 |
| 10 | exp RNA, Viral/ | 89967 |
| 11 | exp Virus Replication/ | 90821 |
| 12 | ((hiv or hiv-1 or vir*) adj (rna or ribonucleic acid)).tw. | 35211 |
| 13 | ((viral or virus or virologic*) adj (load* or replication* or response* or suppression or failure)).tw. | 110989 |
| 14 | 9 or 10 or 11 or 12 or 13 | 263366 |
| 15 | Afghanistan/ or Burundi/ or Burkina Faso/ or Central African Republic/ or "Democratic Republic of the Congo"/ or Eritrea/ or Ethiopia/ or Gambia/ or Guinea-Bissau/ or Liberia/ or Madagascar/ or Mali/ or Mozambique/ or Malawi/ or Niger/ or "Democratic People's Republic of Korea"/ or Rwanda/ or Sudan/ or Sierra Leone/ or Somalia/ or South Sudan/ or Syria/ or Chad/ or Togo/ or Uganda/ or Yemen/ | 94146 |
| 16 | (Afghanistan or Burundi or Burkina Faso or Central African Republic or "Democratic Republic of the Congo" or Eritrea or Ethiopia or Gambia or Guinea-Bissau or Liberia or Madagascar or Mali or Mozambique or Malawi or Niger or "Democratic People's Republic of Korea" or Rwanda or Sudan or Sierra Leone or Somalia or South Sudan or Syria or Chad or Togo or Uganda or Yemen).tw. | 136695 |
| 17 | Angola/ or Benin/ or Bangladesh/ or Bolivia/ or Bhutan/ or Cote d'Ivoire/ or Cameroon/ or Congo/ or Comoros/ or Cabo Verde/ or Djibouti/ or Algeria/ or Egypt/ or Micronesia/ or Ghana/ or Guinea/ or Honduras/ or Haiti/ or India/ or Iran/ or Jordan/ or Kenya/ or Kyrgyzstan/ or Cambodia/ or Kiribati/ or Laos/ or Lebanon/ or Sri Lanka/ or Lesotho/ or Morocco/ or Myanmar/ or Mongolia/ or Mauritania/ or Nigeria/ or Nicaragua/ or Nepal/ or Pakistan/ or Philippines/ or Papua New Guinea/ or Senegal/ or Solomon Islands/ or Eswatini/ or Tajikistan/ or Timor-Leste/ or Tunisia/ or Tanzania/ or Ukraine/ or Uzbekistan/ or Vietnam/ or Vanuatu/ or Samoa/ or Zambia/ or Zimbabwe/ | 456717 |
| 18 | (Angola or Benin or Bangladesh or Bolivia or Bhutan or "Cote d'Ivoire" or Cameroon or Congo or Comoros or "Cabo Verde" or Djibouti or Algeria or Egypt or Micronesia or Ghana or Guinea or Honduras or Haiti or India or Iran or Jordan or Kenya or Kyrgyzstan or Cambodia or Kiribati or Laos or Lebanon or "Sri Lanka" or Lesotho or Morocco or Myanmar or Mongolia or Mauritania or Nigeria or Nicaragua or Nepal or Pakistan or Philippines or "Papua New Guinea" or Senegal or "Solomon Islands" or Eswatini or Tajikistan or "Timor-Leste" or Tunisia or Tanzania or Ukraine or Uzbekistan or Vietnam or Vanuatu or Samoa or Zambia or Zimbabwe).tw. | 626959 |
| 19 | Albania/ or Argentina/ or Armenia/ or Azerbaijan/ or Bulgaria/ or "Bosnia and Herzegovina"/ or Belarus/ or Belize/ or Brazil/ or Botswana/ or China/ or Colombia/ or Costa Rica/ or Cuba/ or Dominica/ or Dominican Republic/ or Ecuador/ or Fiji/ or Gabon/ or Georgia/ or Equatorial Guinea/ or Grenada/ or Guatemala/ or Indonesia/ or Iraq/ or Jamaica/ or Kazakhstan/ or Libya/ or Saint Lucia/ or Moldova/ or Maldives/ or Mexico/ or Marshall Islands/ or North Macedonia/ or Montenegro/ or Mauritius/ or Malaysia/ or Namibia/ or Peru/ or Palau/ or Paraguay/ or Russia/ or El Salvador/ or Serbia/ or Suriname/ or Thailand/ or Turkmenistan/ or Tonga/ or Turkey/ or Tuvalu/ or "Saint Vincent and the Grenadines"/ or Kosovo/ or South Africa/ | 754006 |
| 20 | (Albania or Argentina or Armenia or Azerbaijan or Bulgaria or "Bosnia and Herzegovina" or Belarus or Belize or Brazil or Botswana or China or Colombia or "Costa Rica" or Cuba or Dominica or "Dominican Republic" or Ecuador or Fiji or Gabon or Georgia or "Equatorial Guinea" or Grenada or Guatemala or Indonesia or Iraq or Jamaica or Kazakhstan or Libya or "Saint Lucia" or Moldova or Maldives or Mexico or "Marshall Islands" or "North Macedonia" or Montenegro or Mauritius or Malaysia or Namibia or Peru or Palau or Paraguay or Russia or "El Salvador" or Serbia or Suriname or Thailand or Turkmenistan or Tonga or Turkey or Tuvalu or "Saint Vincent and the Grenadines" or Kosovo or "South Africa").tw. | 796084 |
| 21 | Venezuela/ | 5202 |
| 22 | Venezuela.tw. | 6007 |
| 23 | ("Congo, Dem. Rep." or DRC or The Gambia or "Korea, Dem. People's Rep." or the Democratic People's Republic of Korea or North Korea or Syrian Arab Republic or "Yemen, Rep." or Republic of Yemen or Arab Republic of Egypt or Federated States of Micronesia or Islamic Republic of Iran or Kyrgyz Republic or Lao PDR or "St. Lucia" or Russian Federation or Turkiye or "Türkiye" or "St. Vincent and the Grenadines" or "São Tomé and Príncipe" or "Sao Tome and Principe" or "West Bank and Gaza").tw. | 16560 |
| 24 | 15 or 16 or 17 or 18 or 19 or 20 or 21 or 22 or 23 | 1957457 |
| 25 | 7 and 8 and 14 and 24 | 265 |
| 26 | limit 25 to yr="2019 -Current" | 256 |
| 27 | limit 26 to english | 256 |

Embase Classic+Embase <1947 to 2024 September 27>

| **Step** | **Query** | **Results** |
| --- | --- | --- |
| 1 | exp HIV Infection/ | 836600 |
| 2 | exp HIV/ | 227225 |
| 3 | exp Acquired Immunodeficiency Syndrome/ | 584377 |
| 4 | (HIV or HIV-1 or human immunodeficiency virus or human immuno deficiency virus or human immunedeficiency virus or human immune deficiency virus or AIDS or acquired immunodeficiency syndrome or acquired immuno deficiency syndrome or acquired immunedeficiency syndrome or acquired immune deficiency syndrome).tw. | 614781 |
| 5 | (HIV or HIV-1 or human immunodeficiency virus or human immuno deficiency virus or human immune deficiency virus).tw. | 499812 |
| 6 | (AIDS or acquired immunodeficiency syndrome or acquired immuno deficiency syndrome or acquired immune deficiency syndrome).tw. | 213290 |
| 7 | 1 or 2 or 3 or 4 or 5 or 6 | 1094909 |
| 8 | (dolutegravir* or DTG* or TLD* or Tivicay or JUL or Juluca or dovato or triumeq or soltegravir or GSK1349572 or "S/GSK1349572" or "GSK-1349572" or "GSK 1349572").tw. | 19181 |
| 9 | exp Viral Load/ | 117759 |
| 10 | exp RNA, Viral/ | 94287 |
| 11 | exp Virus Replication/ | 131758 |
| 12 | ((hiv or hiv-1 or vir*) adj (rna or ribonucleic acid)).tw. | 43774 |
| 13 | ((viral or virus or virologic*) adj (load* or replication* or response* or suppression or failure)).tw. | 157293 |
| 14 | 9 or 10 or 11 or 12 or 13 | 354087 |
| 15 | Afghanistan/ or Burundi/ or Burkina Faso/ or Central African Republic/ or "Democratic Republic of the Congo"/ or Eritrea/ or Ethiopia/ or Gambia/ or Guinea-Bissau/ or Liberia/ or Madagascar/ or Mali/ or Mozambique/ or Malawi/ or Niger/ or "Democratic People's Republic of Korea"/ or Rwanda/ or Sudan/ or Sierra Leone/ or Somalia/ or South Sudan/ or Syria/ or Chad/ or Togo/ or Uganda/ or Yemen/ | 133649 |
| 16 | (Afghanistan or Burundi or Burkina Faso or Central African Republic or "Democratic Republic of the Congo" or Eritrea or Ethiopia or Gambia or Guinea-Bissau or Liberia or Madagascar or Mali or Mozambique or Malawi or Niger or "Democratic People's Republic of Korea" or Rwanda or Sudan or Sierra Leone or Somalia or South Sudan or Syria or Chad or Togo or Uganda or Yemen).tw. | 160634 |
| 17 | Angola/ or Benin/ or Bangladesh/ or Bolivia/ or Bhutan/ or Cote d'Ivoire/ or Cameroon/ or Congo/ or Comoros/ or Cabo Verde/ or Djibouti/ or Algeria/ or Egypt/ or Micronesia/ or Ghana/ or Guinea/ or Honduras/ or Haiti/ or India/ or Iran/ or Jordan/ or Kenya/ or Kyrgyzstan/ or Cambodia/ or Kiribati/ or Laos/ or Lebanon/ or Sri Lanka/ or Lesotho/ or Morocco/ or Myanmar/ or Mongolia/ or Mauritania/ or Nigeria/ or Nicaragua/ or Nepal/ or Pakistan/ or Philippines/ or Papua New Guinea/ or Senegal/ or Solomon Islands/ or Eswatini/ or Tajikistan/ or Timor-Leste/ or Tunisia/ or Tanzania/ or Ukraine/ or Uzbekistan/ or Vietnam/ or Vanuatu/ or Samoa/ or Zambia/ or Zimbabwe/ | 664976 |
| 18 | (Angola or Benin or Bangladesh or Bolivia or Bhutan or "Cote d'Ivoire" or Cameroon or Congo or Comoros or "Cabo Verde" or Djibouti or Algeria or Egypt or Micronesia or Ghana or Guinea or Honduras or Haiti or India or Iran or Jordan or Kenya or Kyrgyzstan or Cambodia or Kiribati or Laos or Lebanon or "Sri Lanka" or Lesotho or Morocco or Myanmar or Mongolia or Mauritania or Nigeria or Nicaragua or Nepal or Pakistan or Philippines or "Papua New Guinea" or Senegal or "Solomon Islands" or Eswatini or Tajikistan or "Timor-Leste" or Tunisia or Tanzania or Ukraine or Uzbekistan or Vietnam or Vanuatu or Samoa or Zambia or Zimbabwe).tw. | 841592 |
| 19 | Albania/ or Argentina/ or Armenia/ or Azerbaijan/ or Bulgaria/ or "Bosnia and Herzegovina"/ or Belarus/ or Belize/ or Brazil/ or Botswana/ or China/ or Colombia/ or Costa Rica/ or Cuba/ or Dominica/ or Dominican Republic/ or Ecuador/ or Fiji/ or Gabon/ or Georgia/ or Equatorial Guinea/ or Grenada/ or Guatemala/ or Indonesia/ or Iraq/ or Jamaica/ or Kazakhstan/ or Libya/ or Saint Lucia/ or Moldova/ or Maldives/ or Mexico/ or Marshall Islands/ or North Macedonia/ or Montenegro/ or Mauritius/ or Malaysia/ or Namibia/ or Peru/ or Palau/ or Paraguay/ or Russia/ or El Salvador/ or Serbia/ or Suriname/ or Thailand/ or Turkmenistan/ or Tonga/ or Turkey/ or Tuvalu/ or "Saint Vincent and the Grenadines"/ or Kosovo/ or South Africa/ | 919519 |
| 20 | (Albania or Argentina or Armenia or Azerbaijan or Bulgaria or "Bosnia and Herzegovina" or Belarus or Belize or Brazil or Botswana or China or Colombia or "Costa Rica" or Cuba or Dominica or "Dominican Republic" or Ecuador or Fiji or Gabon or Georgia or "Equatorial Guinea" or Grenada or Guatemala or Indonesia or Iraq or Jamaica or Kazakhstan or Libya or "Saint Lucia" or Moldova or Maldives or Mexico or "Marshall Islands" or "North Macedonia" or Montenegro or Mauritius or Malaysia or Namibia or Peru or Palau or Paraguay or Russia or "El Salvador" or Serbia or Suriname or Thailand or Turkmenistan or Tonga or Turkey or Tuvalu or "Saint Vincent and the Grenadines" or Kosovo or "South Africa").tw. | 1008328 |
| 21 | Venezuela/ | 7757 |
| 22 | Venezuela.tw. | 7718 |
| 23 | ("Congo, Dem. Rep." or DRC or The Gambia or "Korea, Dem. People's Rep." or the Democratic People's Republic of Korea or North Korea or Syrian Arab Republic or "Yemen, Rep." or Republic of Yemen or Arab Republic of Egypt or Federated States of Micronesia or Islamic Republic of Iran or Kyrgyz Republic or Lao PDR or "St. Lucia" or Russian Federation or Turkiye or "Türkiye" or "St. Vincent and the Grenadines" or "São Tomé and Príncipe" or "Sao Tome and Principe" or "West Bank and Gaza").tw. | 27144 |
| 24 | 15 or 16 or 17 or 18 or 19 or 20 or 21 or 22 or 23 | 2385911 |
| 25 | 7 and 8 and 14 and 24 | 495 |
| 26 | limit 25 to yr="2019 -Current" | 455 |
| 27 | limit 26 to english | 454 |

# **Appendix S4. Risk of Bias Assessment for Included Studies**

The risk of bias in the included studies was systematically evaluated using the Joanna Briggs Institute Critical Appraisal Tools for cohort and prevalence studies (<https://jbi.global/critical-appraisal-tools>).

For the cohort studies, certain criteria, such as group comparability and exposure measurement, were excluded from the assessment as they were not relevant to the study objectives. **Table S4.1** provides an overview of the risk of bias assessment for the included cohort studies. All studies demonstrated valid and reliable outcome measurements, adequate follow-up durations, and generally appropriate statistical analyses. While insufficient strategies to address confounding factors were identified as a common issue, these were not considered critical sources of bias, as this review focused on prevalence estimates rather than measures of association.

**Table S4.2** summarises the risk of bias for the included cross-sectional studies. Overall, the studies generally had an appropriate sample frame and used valid methods to identify and measure the condition of interest, along with adequate statistical analyses. However, two of the three studies had unclear or inadequate sampling methods and sample sizes, potentially limiting the generalisability of their findings.

**Table S4.1: Summary of Risk of Bias Assessment for Cohort Studies in the Systematic Review**

| **Study ID** | **Were confounding factors identified?** | **Were strategies to deal with confounding factors stated?** | **Were the outcomes measured in a valid and reliable way?** | **Was the follow up time reported and sufficient to be long enough for outcomes to occur?** | **Was follow up complete, and if not, were the reasons to loss to follow up described and explored?** | **Were strategies to address incomplete follow up utilized?** | **Was appropriate statistical analysis used?** |
| --- | --- | --- | --- | --- | --- | --- | --- |
| McCluskey 2024 a | Yes | Yes | Yes | Yes | Yes | No | Yes |
| Skrivankova 2024 | Yes | Yes | Yes | Yes | No | No | Yes |
| Brites 2022 | Unclear | No | Yes | Yes | Yes | Yes | Yes |
| Avalos 2019 | No | No | Yes | Yes | No | No | Yes |
| Kityo 2022 | No | No | Yes | Yes | Yes | No | Yes |
| Marc 2023 | No | No | Yes | Yes | Yes | No | Yes |
| Meireles 2019 | Yes | Yes | Yes | Yes | No | No | Yes |
| Correa 2020 | No | No | Yes | Yes | No | No | Yes |
| Buju 2022 | Yes | Yes | Yes | Yes | Yes | Yes | Yes |
| Schramm 2022 | Yes | Yes | Yes | Yes | Yes | Yes | Yes |
| Patel 2021 | Yes | Yes | Yes | Yes | Unclear | No | Yes |
| Gemechu 2023 | Yes | Yes | Yes | Yes | No | No | Yes |
| Milward 2023 | No | No | Yes | Yes | No | No | Yes |
| Silva 2023 | Yes | Yes | Yes | Yes | Yes | Yes | Yes |
| Gebremedhin 2024 | No | No | Yes | Yes | Yes | No | Yes |
| Kouamou 2024 | Yes | Yes | Yes | Yes | Yes | No | Yes |
| Sengupta 2023 | No | No | Yes | Yes | No | No | Yes |
| Bareng 2024 | Yes | Yes | Yes | Yes | No | Yes | Yes |
| McCluskey 2024 b | Yes | Yes | Yes | Yes | Yes | No | Yes |

*McCluskey 2024 a and McCluskey 2024 b refer to two distinct studies by the same first author published in the same year, conducted in Uganda and South Africa, respectively.*

**Table S4.2: Summary of Risk of Bias Assessment for Cross-Sectional Studies in the Systematic Review**

| **Study ID** | **Was the sample frame appropriate to address the target population?** | **Were study participants sampled in an appropriate way?** | **Was the sample size adequate?** | **Were the study subjects and the setting described in detail?** | **Was the data analysis conducted with sufficient coverage of the identified sample?** | **Were valid methods used for the identification of the condition?** | **Was the condition measured in a standard, reliable way for all participants?** | **Was there appropriate statistical analysis?** | **Was the response rate adequate, and if not, was the low response rate managed appropriately?** |
| --- | --- | --- | --- | --- | --- | --- | --- | --- | --- |
| Semengue 2022 | Unclear | Unclear | Unclear | Yes | Yes | Yes | Yes | Yes | Yes |
| Mehari 2021 | Yes | Yes | Yes | Yes | Yes | Yes | Yes | Yes | Yes |
| Mahale 2023 | Yes | No | Unclear | No | Unclear | Yes | Yes | Yes | Unclear |

# **Appendix S5. GRADE Assessment**

**Table S5.1: Summary of Findings for 12-Month Viral Suppression Outcomes**

| **Category** | **12-month viral suppression: on-treatment** | **12-month viral suppression: intention-to-treat** |
| --- | --- | --- |
| Number of cohorts studied | 14 | 6 |
| Participants with suppression | 39,272 | 13,681 |
| Total participants | 40,055 | 15,131 |
| Pooled estimate (95% CI) | 96% (94–98%) | 89% (82–93%) |
| I² statistic | 97% | 95% |
| Prediction interval | 72–100% | 56–98% |

**Table S5.2: GRADE Domain-Level Assessment for 12-Month Viral Suppression Outcomes**

| **GRADE Domain** | **12-month viral suppression: on-treatment** | **12-month viral suppression: intention-to-treat** |
| --- | --- | --- |
| Risk of bias | Not downgraded: Most cohort studies had low risk of bias, with valid outcome measurement and appropriate analysis. Some had unclear reporting on confounding control or incomplete follow-up, but these limitations were not considered critical for estimating prevalence. Two of three cross-sectional studies had methodological concerns in sampling and reporting; however, all used valid outcome definitions and statistical methods, and their findings were consistent with the overall pooled estimates | Not downgraded: All studies used valid outcome measurement and appropriate analysis. Some lacked detail on strategies to address loss to follow-up, and one had unclear confounding control. These limitations were not judged to critically affect the estimation of viral suppression prevalence |
| Inconsistency | Downgraded: High I² (97%) and wide prediction interval | Downgraded: High I² (95%) and wide prediction interval |
| Indirectness | Not downgraded: Studies applicable to population of interest | Not downgraded: Studies applicable to population of interest |
| Imprecision | Not downgraded: Narrow 95% CI (94–98%) and large sample | Downgraded: Wider 95% CI (82–93%), smaller sample |
| Publication bias | Not assessed due to high heterogeneity | Not assessed due to high heterogeneity |
| Overall certainty | Moderate | Moderate |
